# Serum Adiponectin, Resistin, and Circulating Soluble Receptor for Advanced Glycation End Products in Colectomy Patients

**DOI:** 10.1155/2011/916807

**Published:** 2011-09-07

**Authors:** Theodor Asgeirsson, Sen Zhang, Sok Kean Khoo, James H. Resau, Nadav Dujovny, Anthony J. Senagore

**Affiliations:** ^1^Spectrum Health Research, Spectrum Health, 25 Michigan Avenue NE, Grand Rapids, MI 49503, USA; ^2^Department of Colorectal Surgery, Spectrum Health, 4100 Lake Drive NE, Suite 205, Grand Rapids, MI 49546, USA; ^3^Laboratory of Microarray Technology, Van Andel Research Institute, 333 Bostwick Avenue NE, Grand Rapids, MI 49503, USA; ^4^Laboratory of Analytical, Cellular, and Molecular Microscopy, Van Andel Research Institute, 333 Bostwick Avenue NE, Grand Rapids, MI 49503, USA; ^5^Division of Colorectal Surgery, Department of Surgery, Keck School of Medicine, USC, 1441 Eastlake Avenue, Suite 7418, Los Angeles, CA 90033-4612, USA

## Abstract

*Aim*. Surgical trauma and associated complications are frequently related to physiological stress during colectomy. This study evaluated the response of adiponectin, resistin, and circulating soluble receptor for advanced glycation end products (sRAGE) in colectomy patients with or without an enhanced recovery protocol. *Method*. Serum samples were collected from 44 colectomy patients at 3 timframes. The surgical procedures were laparoscopic (LAP), hand-assisted laparoscopic (HALS), or open colectomy (OPEN). Adiponectin, resistin, and sRAGE levels were determined by ELISA. Repeated measures ANOVA was applied and *P* values < 0.05 were considered significant. *Results*. A total of 132 (44 × 3) sera were used for analysis. Levels of adiponectin was significantly decreased between PREOP and POD3 (*P* < 0.001). Conversely, concentrations of resistin significantly increased from PREOP to POD1 and returned to baseline value by POD3 (*P* < 0.001). Serum sRAGE levels were significantly higher in LAP in comparison with HALS (*P* = 0.004) and OPEN (*P* < 0.001). sRAGE levels were significantly higher in sera of patients that underwent ERP (*P* < 0.001). *Conclusions*. Serum adiponectin, resistin, and sRAGE have the potential to develop into a panel of stress markers. Higher sRAGE levels in sera of LAP and ERP patients may be indicative of a protective and syngeristic role for colectomy recovery.

## 1. Introduction

**Figure 1:**
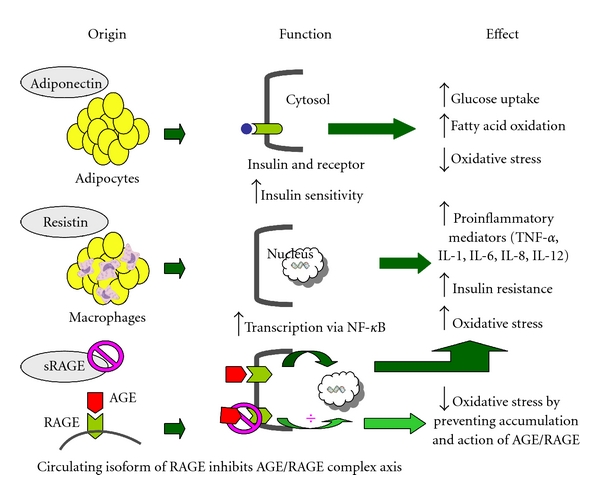
Adiponectin, resistin, and sRAGE as inflammatory/metabolic mediators in human cells.

Perioperative morbidity is particularly frequent with colectomy and results in additional financial burden for the patients as well as the health care systems. Colectomy is also associated with significant physiological stress as a result of the surgical procedure itself, which may impact the risk of complications. Traditionally, the physiological stress in response to surgery has been measured in various ways, such as invasiveness of procedure, length of procedure, and intraoperative blood loss. Hemodynamic changes, peripheral insulin resistance, and the resultant hyperglycemia are also well-known components of surgical stress. However, it remains a challenge to elucidate the specifics of the molecular mediators of the physiological stress response from colectomy. The complexity results from the comprehensive network of cytokines and endocrine mediators that either directly or indirectly impact nitric oxide homeostasis and endothelial function, and the overall inflammatory responses to colectomy [1]. Therefore, investigation of these mediators as stress markers can be crucial for both pre- and postoperative risk assessment in colectomy patients. Adiponectin, resistin, and circulating soluble receptor for advanced glycation end products (sRAGE) (Figure 1) are among those inflammatory and metabolic mediators that may impact preoperative risk assessment and oxidative stress due to surgical trauma. 

Adiponectin is an adipocyte-derived hormone which is secreted into circulating blood and regulates systemic metabolism. Adiponectin has been shown to improve whole-body insulin sensitivity in animal models and diet-induced obesity [2–4]. It stimulates fatty acid oxidation and glucose uptake in skeletal muscle by increasing peripheral insulin sensitivity [5]. Few studies have looked at adiponectin as a potential acute-phase mediator after surgery, which would be important as it also regulates biological processes such as apoptosis, proliferation, migration, and inflammation [6]. 

Resistin was originally reported in a murine model as an adipose-tissue-specific hormone. Further studies have shown that in humans the majority of resistin is derived from macrophages (nonfat stroma fraction of adipose tissue) and, to a lesser extent, from adipocytes, which explain various results in relation to insulin resistance in mouse and human [7, 8]. Resistin promotes a proinflammatory response by modulating nuclear factor kappa-light-chain-enhancer of activated B cells (NF-*κ*B). A recent study has shown resistin as a marker for acute inflammatory response and possibly a prognostic marker in non-sepsis critically ill patients. Correlation was found between HOMA-IR (homeostasis model assessment of insulin resistance) and resistin [9]. Thus, resistin plays an important role in inflammation as well as insulin resistance. 

RAGE or receptor for advanced glycation end products is a multi-ligand receptor that binds to various proteins of different structures. Its promoter contains multiple transcriptional factor-binding sites, such as for NF-*κ*B and other pro-inflammatory genes [10]. sRAGE is a circulating soluble isoform of RAGE, and by competing with RAGE for ligand binding (act as a decoy), it may neutralize RAGE-mediated damage [11]. sRAGE levels have been found to be decreased in several chronic inflammatory diseases [12] however its role as an important marker for inflammatory response during colectomy has not been explored.

In the last 20 years, progress has been made in reducing various complications related to colectomy by standardizing processes of care. Minimal invasive surgery and enhanced recovery programs (ERPs) have played major roles in this transition. Laparoscopy has been shown to decrease length of stay and quicken recovery in colectomy patients, in association with decreased responses in acute inflammatory phase proteins (e.g., CRP and IL-6) [13, 14]. These benefits have been related to less direct stress with smaller wounds and less bowel manipulation. ERP has revolutionized care in colon and rectal surgery. The traditional programs of colorectal operative care have been challenged with successful implementation of ERP without increase in short term morbidity [15, 16]. Although a variety of inflammatory and metabolic mediators have been assessed in laparoscopic and open colectomy patients the specific mediators used in this study have yet to be studied. In addition, there is little assessment of the impact of enhanced recovery program (ERP) programs on these mediators. In this study, we evaluated the patterns of adiponectin, resistin, and sRAGE in patients sera at pre-operative day (PREOP), post-operative day 1 (POD1) and 3 (POD3) with laparoscopic (LAP), hand-assisted laparoscopic surgery (HALS), or open colectomy (OPEN), and with or without ERP, to determine their potentials as stress markers during colectomy.

## 2. Materials and Methods

### 2.1. Patients and Samples Collection

Serum samples for 44 colectomy patients were collected at three time points: pre-operative (PREOP), postoperative day 1 (POD1), and post-operative day 3 (POD3). Whole blood was collected in 5 mL EDTA vials, centrifuged at 3,000 rpm for 10 minutes, and serum was aliquoted and stored at −80°C. All patients were chosen randomly and were blinded to the investigators. Age, gender, body mass index (BMI), anesthesia risk (ASA), operating time, and estimated blood loss (EBL) were also collected. In addition, patients were categorized into 2 groups—compliance or non-compliance with ERP. Noncompliance ERP was defined as i.v. narcotic use lasting more than 48 hours after procedure without other complicating factors of hospital stay. Each participant gave their consent, and this study was approved by the Institutional Review Board committee.

### 2.2. ELISA Assays

Serum adiponectin, resistin, or sRAGE levels were determined by enzyme-linked immuno-absorbent assay (ELISA) according to the manufacturer's protocols (BioVendor LLC, Candler, NC, USA). The sensitivity of each assay was 1 ng/mL, 1 ng/mL and 100 pg/mL, respectively. Each sample analysis was performed in duplicates. 

### 2.3. Statistical Analysis

Data analysis was performed using SSPS windows version 13.0 software (SPSS Inc. Chicago, Ill, USA). Repeated measures ANOVA were used to compare adiponectin, resistin, and sRAGE values between days, operative techniques, and compliance or noncompliance with ERP. *P* values < 0.05 were considered statistically significant. 

## 3. Results

We collected serum samples from 22 LAP, 13 HALS, and 9 OPEN patients, with or without ERP. There were 26 males and 18 females, and the mean age was 59.7 ± 13.5 years. Mean length of stay was 5.3 ± 1.9 days. The most common operation was segmental colectomy (*n* = 33; 75%). Clinical features of the colectomy patients are shown in Table 1. A total of 132 (44 × 3) patient's blood samples from PREOP, POD1, and POD3 were used for ELISA assays.

**Figure 2:**
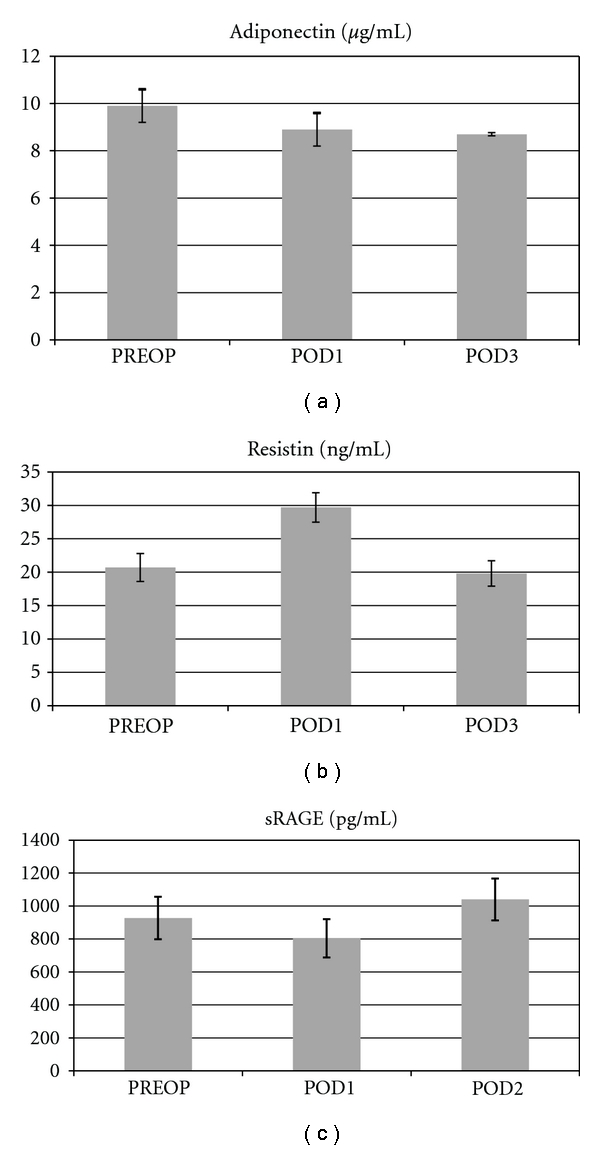
Mean values of serum adiponectin, resistin, and sRAGE concentrations. PREOP, preoperative day; POD1, postoperative day 1; POD3, postoperative day 3.

OR time, BMI, ASA, EBL, and gender did not affect the values of adiponectin, resistin, and sRAGE. During the investigation period, adiponectin levels were significantly decreased between PREOP and POD3 (*P* < 0.001) (Figure 2(a)). The serum concentrations of resistin were significantly increased from PREOP to POD1 and reached baseline value by POD3 (*P* < 0.001) (Figure 2(b)). Average resistin value was higher in patients 75 years of age and older (26.9 versus 19.9 *μ*g/mL) (*P* < 0.001). 

**Figure 3:**
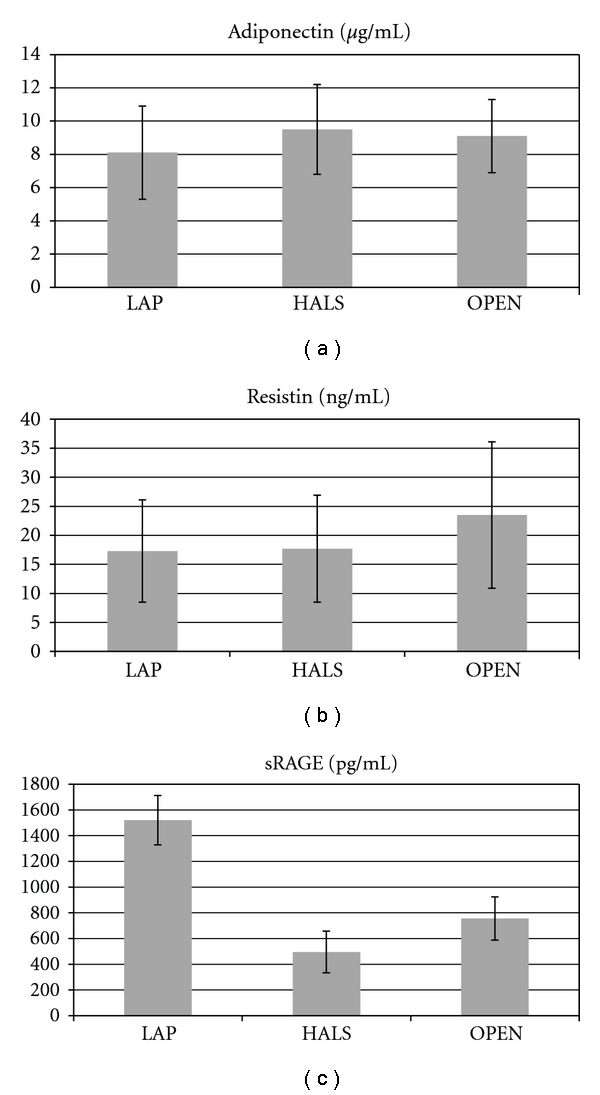
Mean values of serum adiponectin, resistin, and sRAGE. LAP, laparoscopy; HALS, hand-assisted laparoscopy; OPEN, open colectomy.

**Figure 4:**
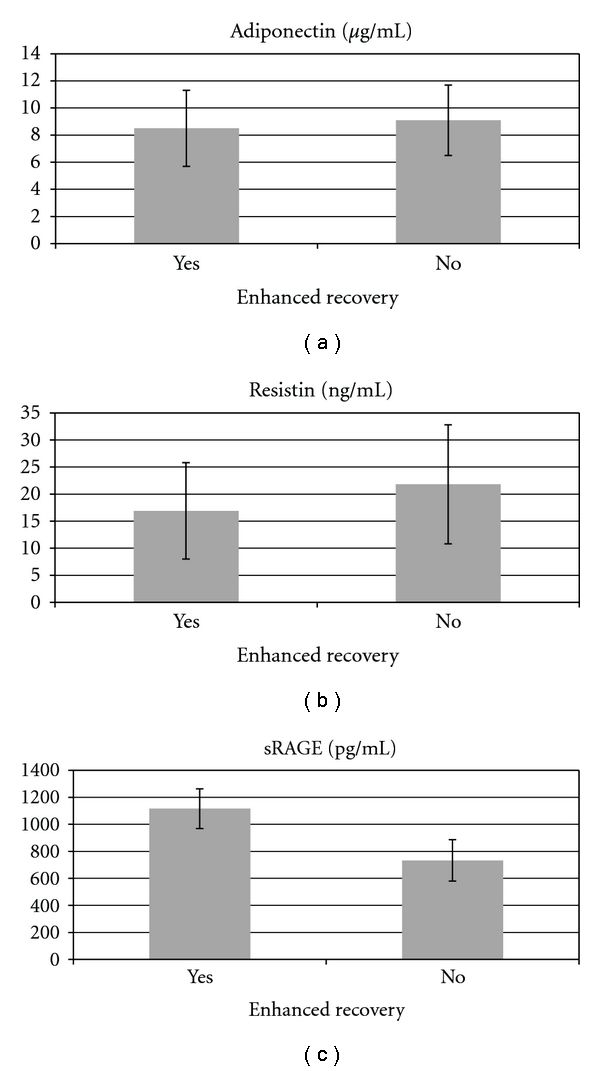
Mean values of serum adiponectin, resistin, and sRAGE.

There was no significant difference of both adiponectin and resistin levels among patients with LAP versus HAL versus OPEN (Figures 3(a) and 3(b). However, the serum sRAGE levels were significantly higher in LAP procedure in comparison with HALS (*P* = 0.004) and OPEN (*P* < 0.001) (Figure 3(c)). sRAGE levels were also significantly higher in sera of patients that underwent ERP (*P* < 0.001) (Figure 4(c)). 

## 4. Discussion and Conclusions

Inflammatory/endocrine cytokines and mediators can have various effects on cellular function through a cascade of pathways that effect oxidative stress and endothelial function. This cascade may lead to organ dysfunction that can be detectable through physiologic changes related to known disease entities like the metabolic syndrome, arthritic, diseases and cardiovascular disorders. Some mediators/cytokines may be protective (IL-10, IL-4, adiponectin) while others have more detrimental effects (IL-1, TNF-*α*, IL-6). Thus, elucidating cytokine and mediator responses before, during, and after surgical procedures may point to predictors for pre- and postoperative risk in colectomy patients. 

Adiponectin has been known as an important regulator for glucose metabolism, as well as having protective anti-inflammatory properties. In our study, serum adiponectin levels decreased significantly from PREOP to POD3. During colectomy, reducing level of adiponectin may result in a systemic response which increases the synthesis of proinflammatory cytokines. Thus, adiponectin can be a marker indicative of a significant degree of surgical trauma. On the other hand, adiponectin levels have been reported to correlate inversely with insulin resistance [17]. The potential exists that the decreasing adiponectin levels (PREOP to POD3) observed in this study could be related to the frequently identified postoperative insulin-resistance-induced hyperglycemia in colectomy patients. It will be interesting to investigate the serum adiponectin levels of hyperglycemic colectomy patients to assess serum adiponectin's potential as a predictor of progression to insulin resistance. 

Critical care patients have demonstrated elevated resistin levels, particularly in association with sepsis. These levels showed an association with other inflammatory markers, including white blood cell count, CRP, and procalcitonin, and with the proinflammatory cytokines IL-6, IL-10, and TNF-*α* [9]. Resistin levels have also been correlated to hyperinsulinemia, hyperglycemia, and insulin resistance in several studies [18–20]. Here, we showed that serum resistin levels increased significantly from PREOP to POD1 and returned to baseline value in POD3 in colectomy patients. Increased resistin levels may be an additional mechanism for the insulin resistance and inflammatory response caused by surgery. 

It has been reported that sRAGE level is downregulated in plasma of chronic hyperglycemic patients [21]. Thus, the initial nonsignificant decreased level of sRAGE may be associated with the increment of insulin resistance indicated by the adiponectin and resistin levels. On the other hand, we found that serum sRAGE levels were significantly higher in patients with LAP procedure in comparison with HALS and OPEN which may be a further explanation for the reduction in complications typically associated with minimally invasive colectomy. sRAGE levels were also significantly higher in sera of patients that underwent ERP. It is interesting that neither adiponectin nor resistin levels were significantly impacted by operative technique (LAP, HALS, and OPEN), or by the use of ER. It is known that sRAGE acts as an endogenous inhibitor of RAGE, neutralizing RAGE-mediating damages by decreasing the expression of matrix metalloproteinases and proinflammatory cytokines TNF-*α* and IL-6 [22]. Therefore, sRAGE is capable of promoting a protective role against the development of inflammation. Higher sRAGE levels which provide higher protection against inflammation may explain the faster perioperative recovery of laparoscopic and ERP patients undergoing colectomy. Thus, sRAGE capacity as a marker for inflammatory stress response and its protective mechanism warrants further investigation. 

This is a proof-of-concept study to suggest that inflammatory and metabolic mediators can be evaluated as potential physiological biomarkers related to surgical stress in colectomy patients. Here, we have demonstrated that serum adiponectin, resistin, and sRAGE have the potential of developing into a panel of operative stress markers for inflammatory response in colectomy patients. Further experimental and clinical studies are required to establish the role of adiponectin and resistin as predictors for insulin resistance progression that is commonly related to surgical complications. Comprehensive and validation studies on other inflammatory and metabolic mediators, besides adiponectin, resistin, and sRAGE presented in this study, will lead to better understanding of the complex immunologic and metabolic responses in colectomy patients which will facilitate risk assessment and improve colectomy management and outcome.

## Figures and Tables

**Table 1:** Clinical data of colectomy patients.

Characteristics	LAP (*n* = 22)	HALS (*n* = 13)	OPEN (*n* = 9)
Age (year, mean ± SD)	64.3 ± 12.6	56.8 ± 13.2	52.4 ± 13.1
Gender			
Male	17	4	5
Female	5	11	4
ERP			
Yes	19	9	0
No	3	4	9
BMI			
≤30	18	8	4
>30	4	5	5
OR time			
≥3 hours	8	2	3
<3 hours	14	11	6
ASA			
>2	6	3	2
≤2	16	11	7
EBL			
≥300 cc	1	1	4
<300 cc	21	12	5

Lap, laparoscopic; HALS, hand-assisted laparoscopic; OPEN, open colectomy; ERP, enhanced recovery program; BMI, body mass index; OR, operation room; ASA, American Society of Anesthesiologists anesthesia risk; EBL, estimated blood loss.
